# A compact pulsatile simulator based on cam-follower mechanism for generating radial pulse waveforms

**DOI:** 10.1186/s12938-018-0620-3

**Published:** 2019-01-03

**Authors:** Tae-Heon Yang, Gwanghyun Jo, Jeong-Hoi Koo, Sam-Yong Woo, Jaeuk U. Kim, Young-Min Kim

**Affiliations:** 1 Department of Electronic Engineering, Korea National University of Transportation, Chungju-si, Chungbuk Republic of Korea; 2 Department of Mathematical Sciences, KAIST, Daejeon, Republic of Korea; 3 Department of Mechanical and Manufacturing Engineering, Miami University, Oxford, OH USA; 4 Center for Mechanical Metrology, KRISS, Daejeon, Republic of Korea; 5 Future Medicine Division, Korea Institute of Oriental Medicine (KIOM), 1672 Yuseongdaero, Yuseong-gu, Deajeon, 34054 Republic of Korea

**Keywords:** Radial pulsation simulator, Cam, Radial artery pressure waveform, Augmentation index

## Abstract

**Background:**

There exists a growing need for a cost-effective, reliable, and portable pulsation simulator that can generate a wide variety of pulses depending on age and cardiovascular disease. For constructing compact pulsation simulator, this study proposes to use a pneumatic actuator based on cam-follower mechanism controlled by a DC motor. The simulator is intended to generate pulse waveforms for a range of pulse pressures and heart beats that are realistic to human blood pulsations.

**Methods:**

This study first performed in vivo testing of a healthy young man to collect his pulse waveforms using a robotic tonometry system (RTS). Based on the collected data a representative human radial pulse waveform is obtained by conducting a mathematical analysis. This standard pulse waveform is then used to design the cam profile. Upon fabrication of the cam, the pulsatile simulator, consisting of the pulse pressure generating component, pressure and heart rate adjusting units, and the real-time pulse display, is constructed. Using the RTS, a series of testing was performed on the prototype to collect its pulse waveforms by varying the pressure levels and heart rates. Followed by the testing, the pulse waveforms generated by the prototype are compared with the representative, in vivo, pulse waveform.

**Results:**

The radial Augmentation Index analysis results show that the percent error between the simulator data and human pulse profiles is sufficiently small, indicating that the first two peak pressures agree well. Moreover, the phase analysis results show that the phase delay errors between the pulse waveforms of the prototype and the representative waveform are adequately small, confirming that the prototype simulator is capable of simulating realistic human pulse waveforms.

**Conclusions:**

This study demonstrated that a very accurate radial pressure waveform can be reproduced using the cam-based simulator. It can be concluded that the same testing and design methods can be used to generate pulse waveforms for other age groups or any target pulse waveforms. Such a simulator can make a contribution to the research efforts, such as development of wearable pressure sensors, standardization of pulse diagnosis in oriental medicine, and training medical professionals for pulse diagnosis techniques.

## Background

The importance of monitoring artery-related factors such as arterial pressure waveform and pulse wave velocity has steadily increased in the medical science and healthcare fields [1–3]. Among the factors for health monitoring, the radial pressure waveform is a surrogate marker for estimating the central aortic pressure and predicting cardiovascular diseases [4–6]. Thus, in recent years, the need for radial artery monitoring sensors is rapidly increasing in order to measure radial pulsation waveforms, which can vary according to human race, sex, age, and health conditions, such as arterial stiffness [7, 8]. To effectively measure the radial artery pulse waveforms, there have been numerous research studies on flexible and wearable sensing technologies. These studies aimed at developing skin-attachable blood pressure sensors with superior sensing properties along with mechanical flexibility and robustness, thus enabling real-time blood pressure measurement or monitoring. Recently, numerous nanomaterials including nanowires [9], carbon nanotubes [10], polymer nanofibers [11], metal nanoparticles [12], and graphene [13] were tested in the design of wearable blood pressure sensors.

With the rapid increase in the research and development of wearable blood pressure sensors, the demand for securing the measurement accuracy of the wearable sensors has also been increased considerably. The accuracy of blood pressure measurement is critically important for the commercial use of such wearable sensors. In the case of hypertension, a 5-mmHg error in blood pressure measurements may double the number of patients diagnosed with hypertension, or even reduce it by half [14]. Despite the importance of measurement accuracy, few studies exist on the evaluation and improvement of wearable sensors’ measurement accuracy. Ideally, for such studies, clinical trials with large numbers of patients are the best way to examine the accuracy of wearable sensor measurements. However, often times, a large-scale human subject testing is limited due to high cost and time constraints. As an alternative to clinical testing, mechanical simulators capable of accurately regenerating standardized radial pulsation waveforms with a variety of different pulse features can be a good means of investigating and improving the measurement accuracy of wearable sensors.

In addition to a growing demand of pulsatile simulators for calibrating blood pressure sensors, they can make significant contributions to the scientific advancement of Oriental Medicine (OM), such as modernizing or standardization of pulse diagnosis techniques. OM or traditional Chinese medicine is a long-established traditional medical practice in Asia, but it is being widely used nowadays in Western countries in the form of alternative medicines. OM practices include pulse diagnosis, acupuncture, and herbal medicine. The pulse diagnosis is one of the most important diagnostic methods in OM. It is based on the 3-finger technique that sense radial pulses at the terminal region of radial artery on a wrist by index, middle, and ring fingers to diagnose health conditions of internal organs. Unfortunately, the pulse diagnosis technique is ambiguous, and it is not standardized. It depends on the pulse characteristics (intensity, patterns, etc.) and location of the figures. Furthermore, it heavily relies on OM doctors’ subjective experiences. Thus, there exists an urgent need for quantification or standardization of pulse waveforms to modernize and teach the pulse diagnosis. Pulsatile simulators capable of reproducing standardized radial pulse waveforms reliably can play an important role in order to train OM students and professionals and to meet the urgent need.

Currently, several simulators generating blood pressure waveforms have been developed. They are mainly based on a mechanism circulating viscous fluids similar to blood. ViVitro Labs, Inc., developed an endovascular simulator that can generate pulsatile flow and blood pressure waveforms similar to those of the human body [15]. This simulator is characterized as a super pump that generates a pulsating flow, and the generated pulsatile flow passes through a viscoelastic impedance adapter, a pump head, and a compliance chamber to an aortic anatomical model. Lee et al. developed a cardiovascular simulator for studying the depth, the rate, the shape, and the strength of radial pulses [16]. The simulator is comprised of a pulse generating part, a vessel part, and a measurement part. Chang et al. developed a pulse simulator based on a hydraulic control method [17]. The developed simulator can adjust the characteristic parameters of the pulse wave by manipulating the opening time of the hydraulic valve and the hydraulic pressure intensity. Tellyes Scientific, Inc., developed a pulse-training simulator (Victor Pulse) [18]. It was developed to realize 26 pulse waveforms using a method of circulating fluid and opening and closing multiple valves to produce a desired waveform.

Current pulse simulators, including simulators developed in above mentioned studies, are complex, bulky, and expensive. In most simulators, the method of controlling the fluid to generate the blood pressure waveform is a quite complicated. Even if sophisticated and expensive simulators, they have limitations in generating a wide range of radial pulse waveforms, which may be hundreds depending on human race, sex, age, and health conditions, by adjusting several valves and flow rates. Note that it is extremely difficult to control the reflected waves sporadically generated in the liquid. In addition, liquid-based simulators, in particular for portable ones, pose potential problems such as distortion of pressure waveforms due to cavitation and the leakage of liquid.

The primary goal of this study is to develop a cost-effective and portable blood pulse simulator that can accurately and repetitively generate a human radial pulse waveform. To this end, it proposes to use a cam-follower mechanism to generate radial artery waveform. The proposed simulator adopts a pneumatic-driven mechanism to avoid the problem of pressure wave reflection, bubbles, and leakage produced in a liquid-driven device. In this study, a cam profile is determined based on a “standard” radial pulse waveform obtained by in vivo testing of a healthy young man in his 20 s. To demonstration purpose, only one cam is fabricated, but the proposed simulator is designed to easily replace the cam with other types of cams to generate other radial pulse waveforms. The design includes a DC motor connected to the cam and follower mechanism that pushes a piston into a cylinder to simulate the heart beat rate with its speed. The design also includes a “diastolic” chamber to adjust the pulse pressure of the waveforms. Using the prototype simulator, a serious of testing was performed to evaluate its performance in generating radial pulse waveforms. These waveforms were compared with the human pulse profile.

This article is organized as follows. The next section describes the target pulse pressure waveform of the radial artery that the proposed simulator is trying to reproduce. The following design and development section explains the schematic diagram and the developed platform of the radial pulsation simulator based on the physiological behavior of the human body. Finally, after describing the process of the pressure data measurement from the developed simulator, an analysis and discussion of the experimental results conclude the article.

## Methods

### Data collection of human pulse waveforms

**Fig. 1:**
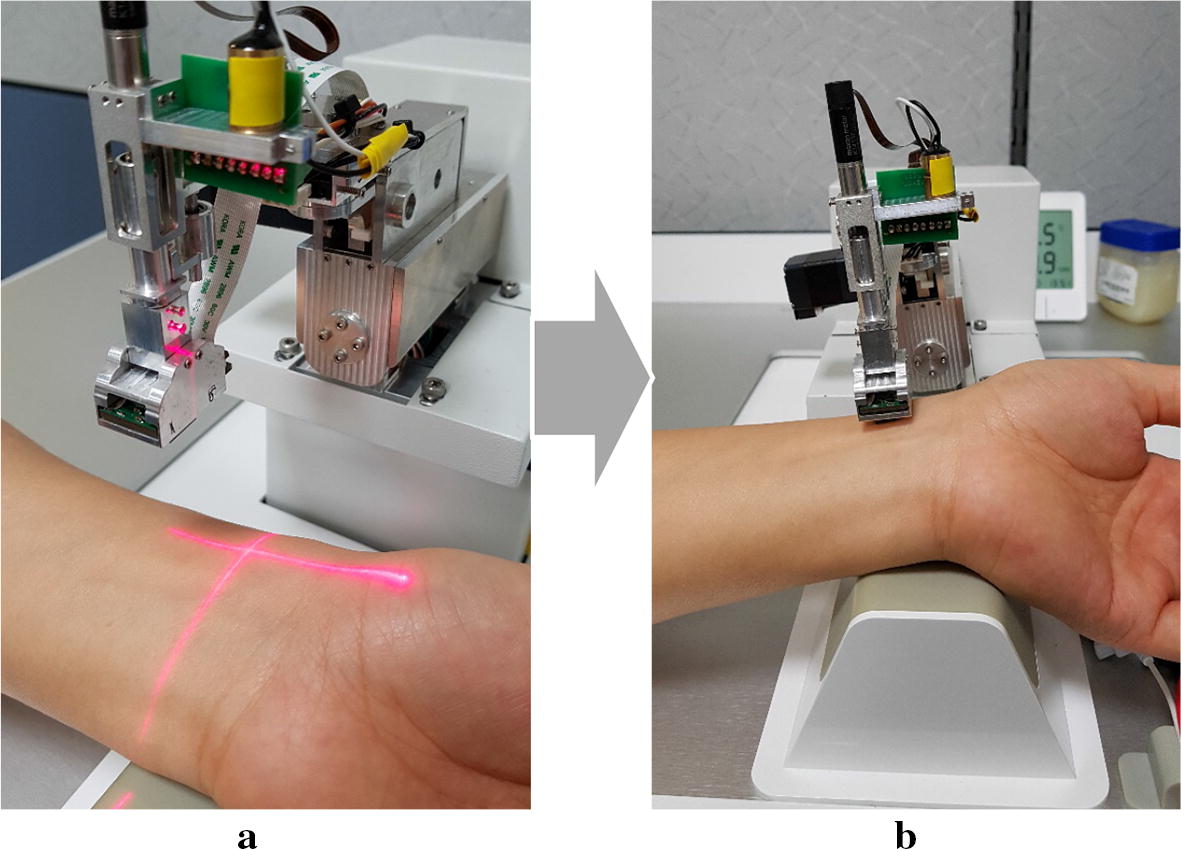
Data collection procedure of human pulse waveforms by the robotic tonometry system: **a** the operator takes the pulsation position with his/her fingers and marks the pulsatile position using the cross shape laser pointer, **b** the RTS detects the appropriate direction and force of the pulse sensor contacting on the wrist skin surface

The reference input signal of a radial pulse waveform was acquired from the clinical data of a healthy young adult because the second peak of the pulse waveform was apparently observed in young people [19, 20]. As shown in Fig. 1, the robotic tonometry system (RTS), developed by the authors at Korea Institute of Oriental Medicine in South Korea, was utilized to obtain the radial pulse waveform with high precision by autonomously detecting the exact pulsation positions and precisely pressurizing the radial artery using a 6-DOF robotic manipulator including one redundant actuator [21]. A pulse sensor array with six pressure sensory channels was attached to the end effector of the RTS to maintain a constant posture and contact force on the radial artery [22]. A 3-DOF motorized stage moved the center position of the pulse sensor to the exact pulsation position. The contact directions between the pulse sensor and the skin surface were controlled by two harmonic-driving actuators without gear backlash. A ball-screw typed linear actuator was used to precisely control the contact force of the pulse sensor.

**Fig. 2:**
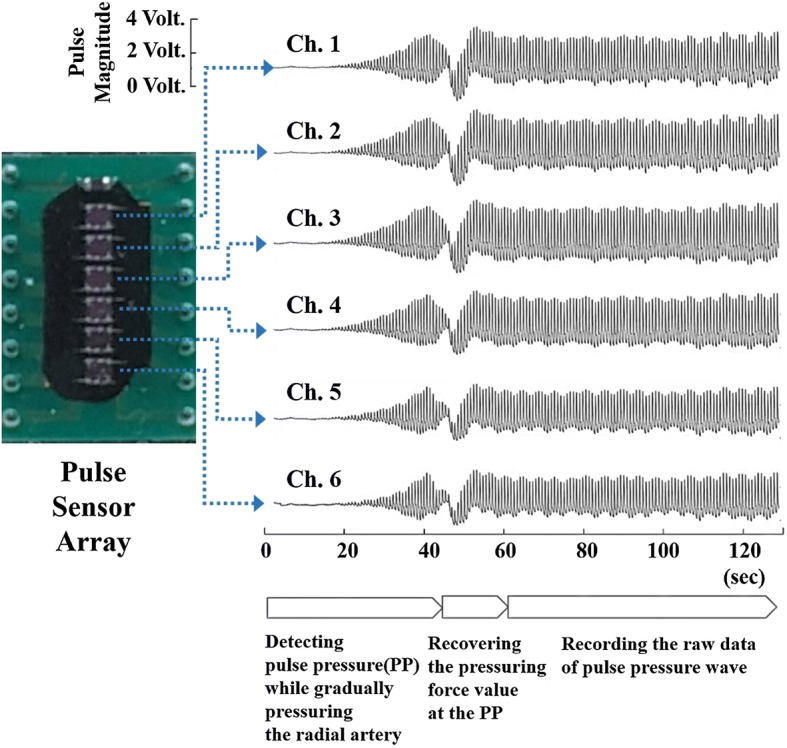
Procedure for detecting the PP and recording the pulse waveform in a reliable state and the raw data acquired from six channels of the pulse sensor array

Figure 2 shows the raw data of the pulse wave measured from the time that the pulse sensor reached for the wrist skin surface to pressurize the artificial radial artery. The pulse sensor incrementally pressed the radial artery until it found the pulse pressure (PP) that was the maximum value of the first peak magnitudes. When the PP was detected, the tonometry device maintained the contact force for about 60 s to reliably record the raw signals of the radial artery pulse waveform at the PP. The final reference signal of the radial artery pulse waveform was obtained by averaging the 40 pulse waveforms recorded in the steady-state region.

### Data processing for obtaining representative waveform

Forty consecutive pulses of the steady state were extracted from the pressure waveforms measured on the wrist of a human subject using the RTS, and the selected pulses were used to generate representative waveforms. In order to calculate the representative waveform effectively, first, each pulse is normalized to have the same period: T = 1. The function representing the normalized 40 pulses and the mean value are defined as ${\text{u}}_{\text{i}} : {\text{T}} \to {\text{R}}$, (i = 1,…, 40) and $\overline{u} : {\text{T}} \to {\text{R}},$ respectively. In the mean value $\overline{u}$, the value of the radial augmentation index (AI), which is the most important statistical value for evaluating the clinically important arterial stiffness, is relatively small, and thus the mean value could not be used as a representative waveform. Therefore, the minimization problem expressed by Eq. (1) using the total error $E\left( {\text{u}} \right)$ was defined to obtain the representative waveform that maximally preserved the value of the radial AI.

1 $$ \begin{aligned} & {\text{Minimize}}\;E\left( {\text{u}} \right) = \sqrt {{\text{E}}_{{{\text{L}}^{2} }} \left( u \right)^{2} + \left( {\alpha E_{RI} \left( u \right)} \right)^{2} } \\ & \quad {\text{in u}} \in {\text{L}}^{2} \left( {\text{T}} \right),\quad {\text{where}} \\ & {\text{L}}^{2} \;{\text{error of total waveform:}}\;E_{{L^{2} }} \left( u \right) = \frac{1}{40}\sqrt {\mathop \sum \limits_{i = 1, \ldots ,40} \mathop \smallint \limits_{T} \left( {u_{i} - u} \right)^{2} } \\ & {\text{Error of radial AI: }}E_{RI} \left( u \right) = \left| {RI - \overline{RI\left( u \right)} } \right|. \\ \end{aligned} $$

Here, $RI$ is the mean value of the radial AI of $u_{i} \left( {i = 1, \ldots ,40} \right)$, and $\overline{RI\left( u \right)}$ is the radial AI of the u function. If the value of α in $E\left( {\text{u}} \right)$ is set to a sufficiently large constant, the solution minimizing $E\left( {\text{u}} \right)$ can preserve the value of the radial AI because Eq. (1) becomes a penalty problem using the radial AI. Thus, the solution obtained by solving the minimization problem, Eq. (1), in the N-dimensional space of the discrete Fourier series function can be used as a representative waveform. To solve Eq. (1), the mean value of the pressure pulse $\overline{u}$ is used as the initial guess after it is represented by a N-dimensional discrete Fourier series function, as shown in Eq. (2). Then, using an iterative method based on a line search, the coefficients $a_{k}$ and $b_{k}$ in Eq. (2) are updated in each iteration step. Here, the dimension of the Fourier series function is set to a sufficiently large value of 10 so that the Fourier series function can accurately form a representative waveform:

2 $$ \overline{u} \left( \theta \right) = {\text{a}}_{0} + \mathop \sum \limits_{k = 1}^{n} \left( {a_{k} \cos \left( {\frac{k}{T}\theta } \right) + b_{k} \sin \left( {\frac{k}{T}\theta } \right)} \right) $$

**Fig. 3:**
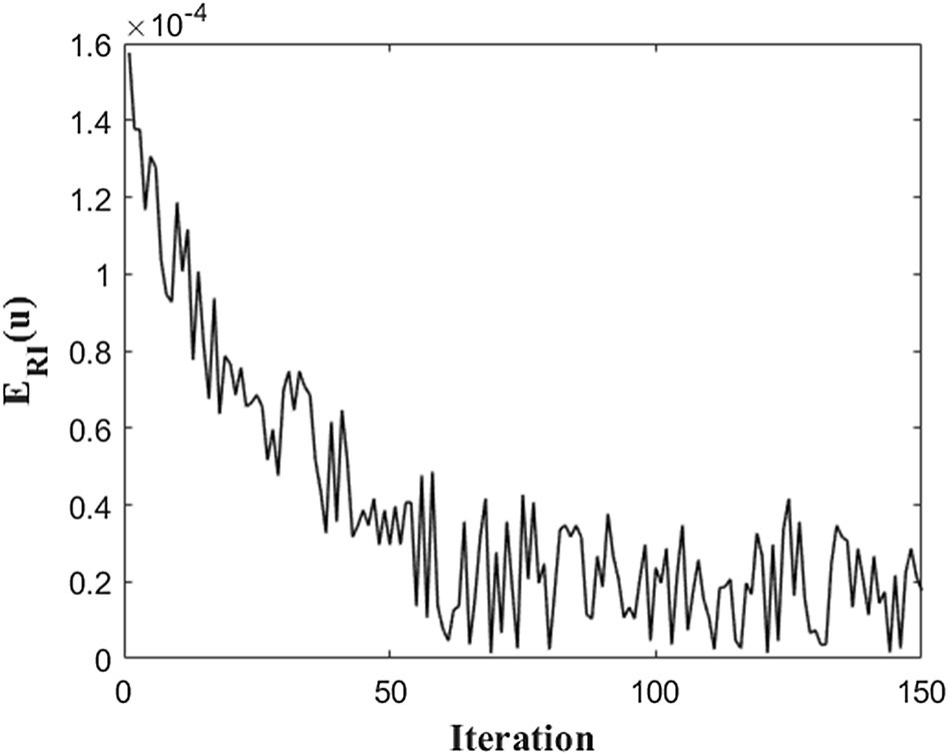
The evolution result of error function of radial AI, $E_{RI} \left( u \right)$ during iteration method

The objective of the minimization problem, Eq. (1), is to find the Fourier series equation that most closely matches the human measured data while minimizing the relative error of the radial AI. Therefore, when the evolution of $E_{RI} \left( u \right)$ is plotted in Fig. 3 while solving the minimization problem using iterative methods. As shown in Fig. 3, 150 iterations were performed so that the relative error of the radial AI could be sufficiently reduced to 0.00247%. Through this iteration process, the coefficients of the Fourier series function of Eq. (2) are obtained as shown in Table 1. In addition, the ${\text{L}}^{2}$ error of the total waveform, $E_{{L^{2} }} \left( u \right)$, is 3.64%, which means that the representative waveform is well matched to the average waveform of $u_{i}$.

**Table 1:** Coefficients of Fourier series

Symbol	Value	Symbol	Value
a0	0.9490		
a1	0.5150	b1	0.5873
a2	0.2893	b2	0.1871
a3	0.2838	b3	0.0459
a4	0.1498	b4	− 0.0032
e5	0.1227	b5	− 0.1109
a6	0.1177	b6	− 0.0743
a7	0.0569	b7	− 0.0196
a8	0.0105	b8	− 0.0086
a9	0.0008	b9	− 0.0043
a10	0.0056	b10	− 0.0007

**Fig. 4:**
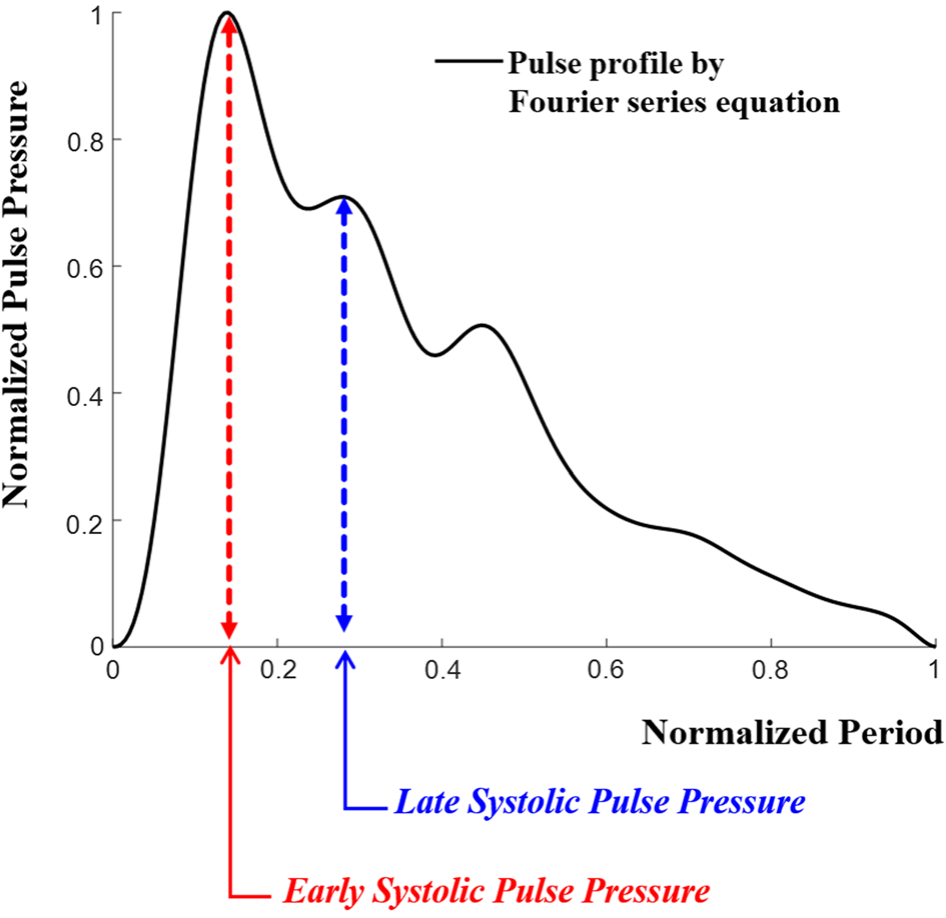
Normalized pulse pressure wave obtained by the Fourier series equations at the time of the normalized period

The coefficients of Table 1 determined through the minimization problem are applied to the representative waveform Eq. (2), and the equation is plotted as shown in Fig. 4. In Fig. 4, the value of the radial augmentation index defined by Eq. (3) is calculated as 73.3%, which is similar to the average radial AI of men (69.5% ± 16.3%, [5]). Therefore, it was confirmed that the representative waveform obtained by the Fourier series preserved the average radial AI of men.

3 $$ Radial\;Augmentation\;Index\;(AI) = \frac{Late\;Systolic\;Pulse\;Pressure}{Early\;Systolic\;Pulse\;Pressure} \times 100\; (\% ) $$

### Fabrication of three-peak cam-based pulsation simulator

**Fig. 5:**
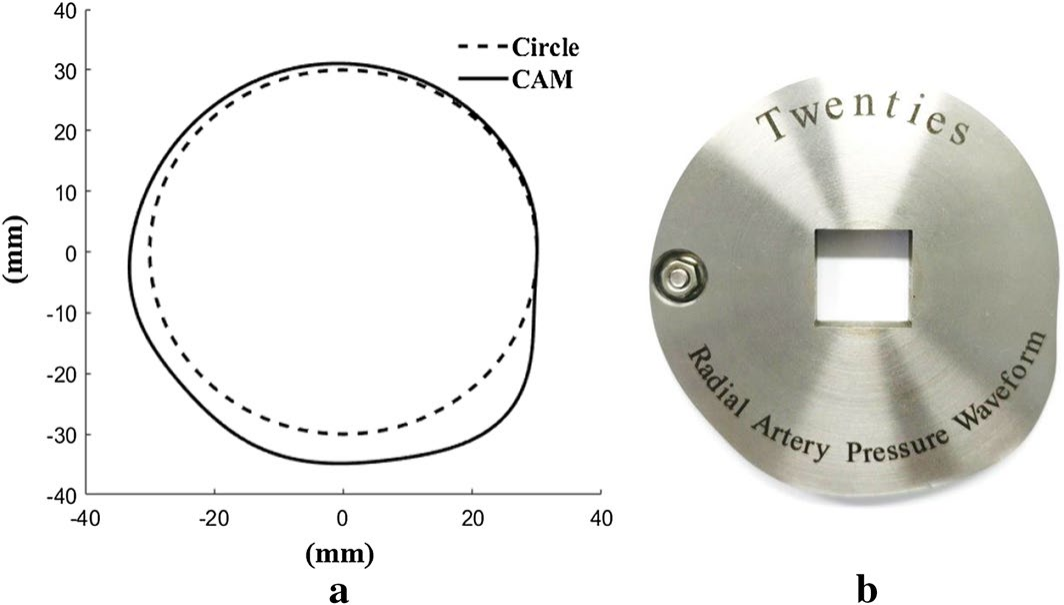
Three-peak cam obtained from the Fourier series equations: **a** three-peak circular cam design, **b** fabricated three-peak cam

In order to convert the Fourier series equations (Fig. 4) obtained from the human data continuously measured by RTS into a three-peak circular shape, the normalized period (Fig. 4) is converted to 360°, and the shape of the cam is schematized as shown in Fig. 5a. This schematized three-peak cam design was fabricated through a wire-cutting machining process of nonmagnetic and high-rigidity material, stainless-steel 304, as shown in Fig. 5b.

**Fig. 6:**
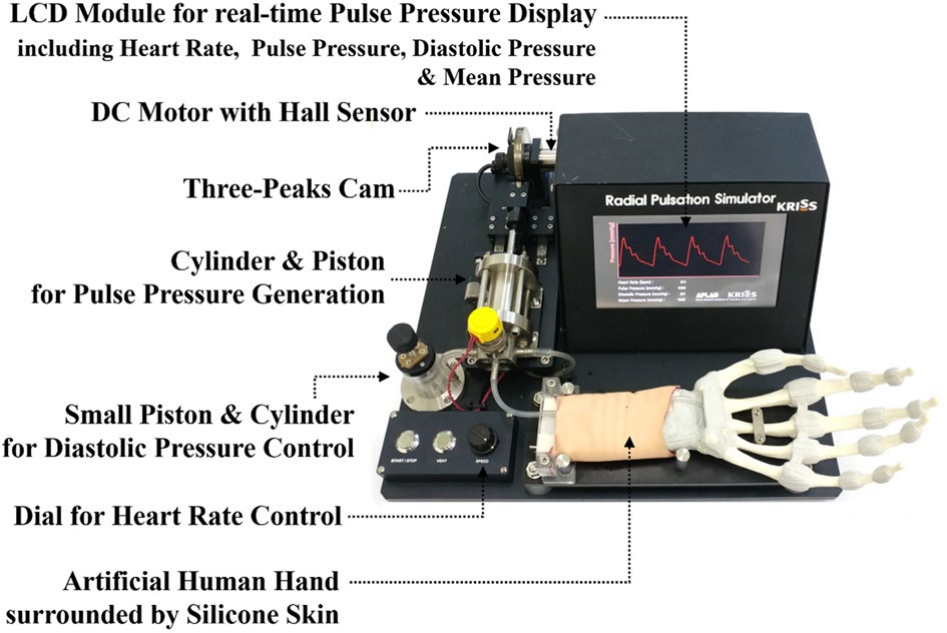
Developed three-peak cam-based affordable pulsation simulator

To design a device capable of regenerating human-like pulse pressure using the fabricated three-peak cam, the cam was mounted on a DC motor (Maxon Motor, DCX 26 L), and a cylinder/piston module capable of repeating compression and tension according to the shape of the cam during rotation was mounted in connection with the cam, as shown in Fig. 6. Here, in order to measure and display the heart rate, a Hall sensor capable of measuring the rotational speed of the DC motor was installed. An additional small cylinder/piston module was installed to control the diastolic pressure by adjusting the amount of air in the cylinder.

In order to facilitate the RTS or human to detect the pulse pressure wave generated when the air in the cylinder is compressed by the piston connected to the cam, a silicon artificial blood vessel was connected to the end of the cylinder, and the blood vessel was supported by an artificial wrist bone for tonometry and was surrounded by silicone skin (3B Scientific, W19310).

To monitor the air pressure inside the cylinder in real time, a small pressure sensor (Honeywell, 40PC006G) was connected to the cylinder by a tube, and the measured pulse pressure value was displayed on the LCD screen in real time. The microprocessor was built in the housing and was used to calculate the pulse pressure, diastolic pressure, and mean pressure from the measured pulse pressure waveform value in real time. These values were displayed on the screen.

## Results

### Evaluation of developed simulator using robotic tonometry system

**Fig. 7:**
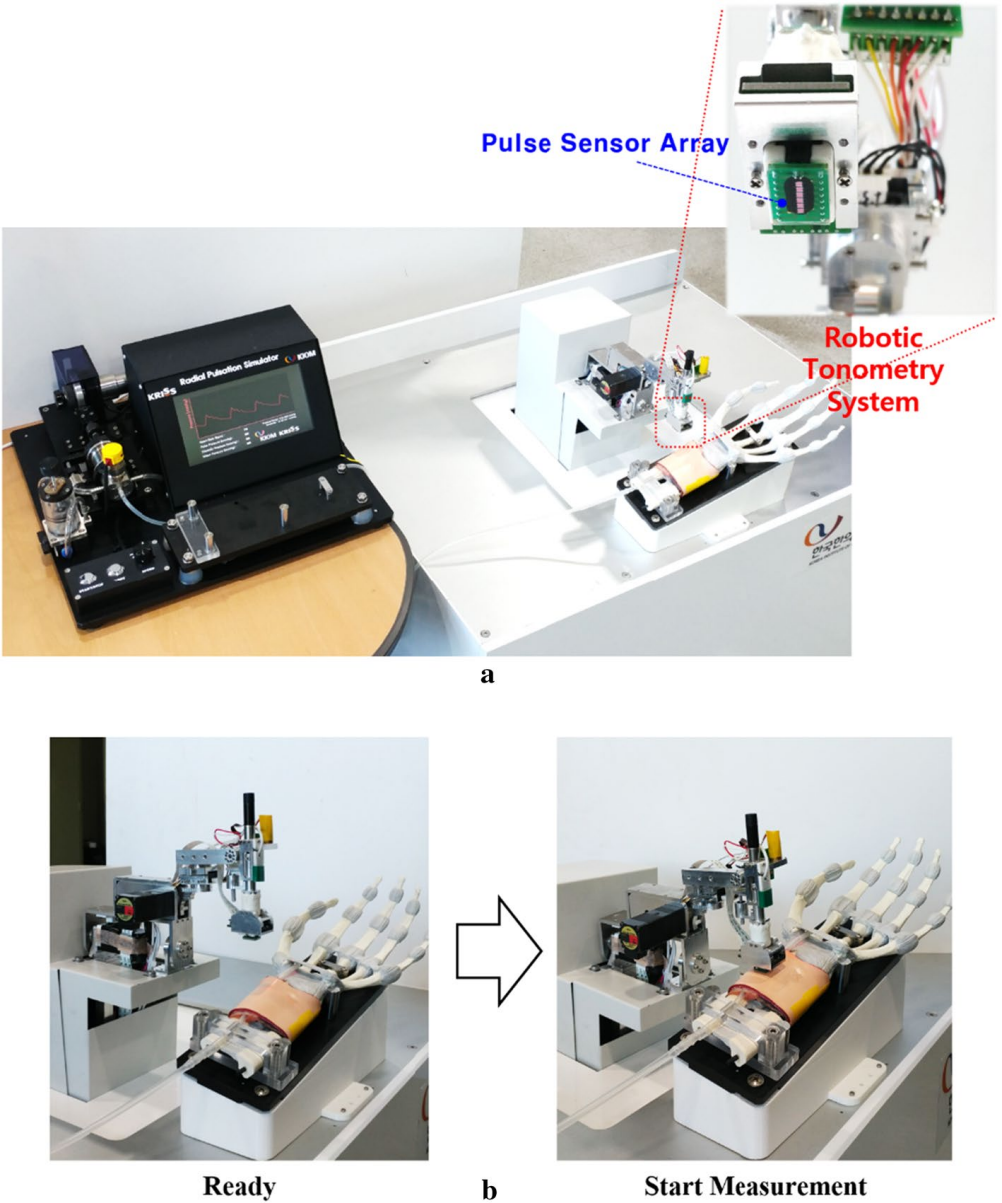
Evaluation of the developed simulator by the robotic tonometry system: **a** measurement setup, **b** measurement procedure

To verify that the developed cam-based pulsation simulator can accurately reproduce the average radial pulse profile of a human measured by RTS, the radial pulse generated at the wrist region of the simulator was measured again using RTS. Figure 7 shows the overall experimental setup for evaluating the developed simulator. The simulator’s wrist part was laid and fixed on the base plate of the RTS similar to the location of the human arm. The developed simulator can modulate the pulse pressure and heart rate generated by the cam-based mechanism by changing the length of the air tube and the rotational speed of the cam. In the experiments, the simulator pulse pressure increased from 50 to 60 mmHg, and the heart rate values increased from 65 to 75 bpm. The generated radial pulse was measured by RTS. While working for 5 min, the simulator showed a repeatability of CV = 0.23% and CV = 0.82% for the heart rate and the pulse pressure, respectively.

**Fig. 8:**
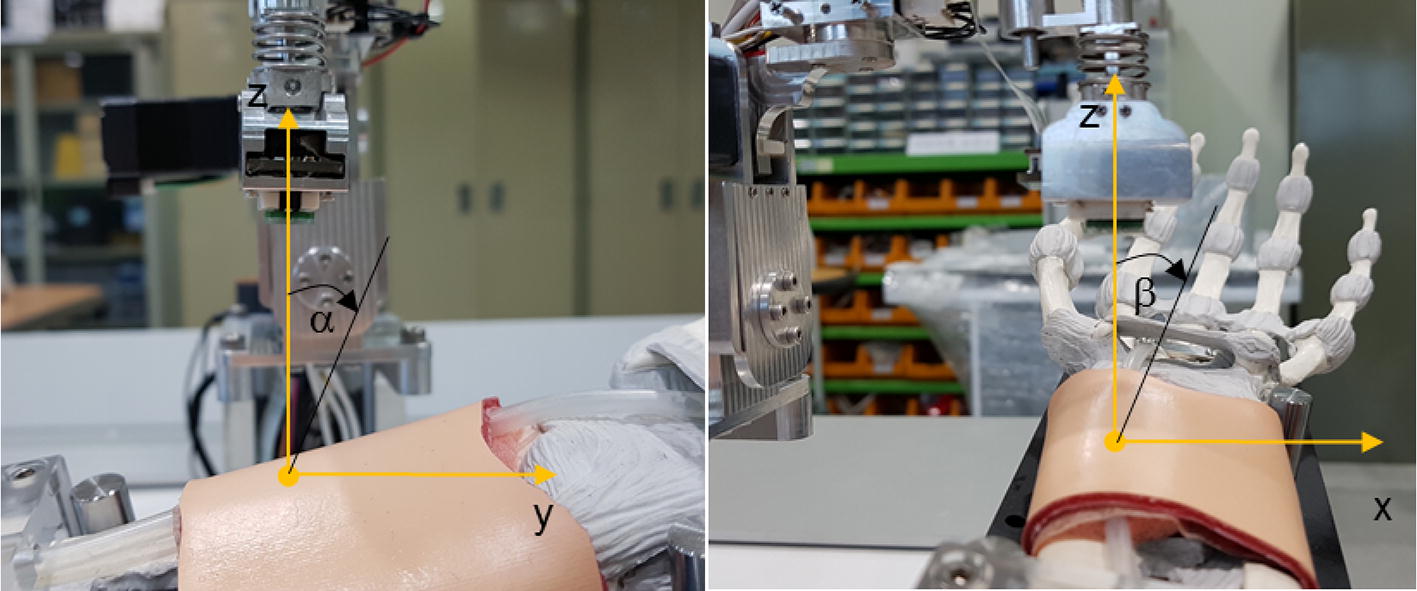
Representation of the definitions of the two vertical contact angles for defining the contact direction with the radial artery

As shown in Fig. 8, since the artificial arm of the simulator was fixed on the base of the robotic tonometry device, the contact force direction of the pulse sensor could be kept constant when the pulse sensor surface angles with the gravitational axis were controlled by the constant target values α = − 5.0° and β = 2.0°. In the experiment, the two contact angles between the pulse sensor surface and the base plane of the simulator were controlled with error bounds of ± 0.21° and ± 0.37°, respectively.

**Fig. 9:**
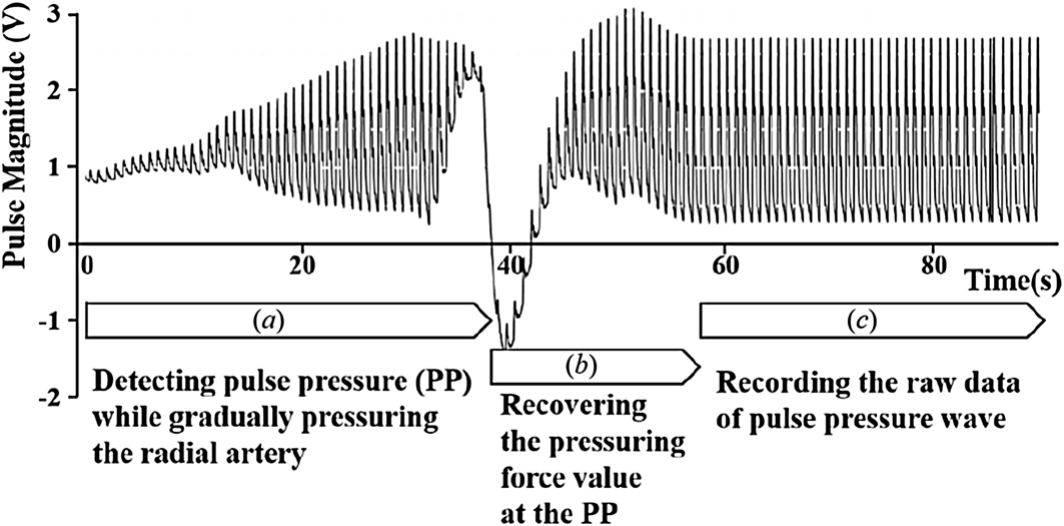
The raw signals of the artificial radial artery pulse wave: **a** detecting the maximum pulse pressure by increasingly pressurizing the radial artery, **b** recovering the pressurizing force values at the maximum pulse pressure, **c** recording the raw data of pulse wave at the maximum pulse pressure

Figure 9 shows the raw data of the pulse wave measured from the time that the pulse sensor reached the artificial wrist surface to the pressurization of the artificial radial artery. The center of the pulse sensor was laid on the same contact point of the surface, and the radial artery was incrementally pressured until the maximum pulse pressure values were found. When the maximum pulse pressure was detected, the tonometry device maintained the contact force for about 30 s to reliably record the raw signals of the maximum pulse pressure of the radial artery pulse. Approximately 30 pulse waveforms obtained in the reliable region were averaged to analyze the dynamic characteristics between the artificial radial pulse and the reference signal obtained from clinical data.

### Analysis

In order to analyze how accurately the developed cam-based pulsation simulator can regenerate the human representative radial pulse waveforms shown in Fig. 4, error analyses were performed among the representative pulse waveforms of the human (Fig. 4), the pulse wave measured by the RTS on the skin above the simulator wrist (Fig. 9), and pressure sensor outputs built into the simulator’s vessel.

First, these error analyses were performed by comparing the radial AI calculated from each radial pressure waveform. This is because the radial AI has a significantly high correlation with the central aortic AI, which is a very important indicator for predicting cardiovascular diseases such as atherosclerosis and vascular aging [5, 23, 24]. Next, these error analyses were also conducted by comparing the phase delay between the first peaks of the representative human pulse wave and the simulator’s measured pulse data.

The first peak of the radial artery pressure waveform was used to reconstruct the early systolic shoulder of the aortic pressure wave through a generalized transfer function [6]. Since the upstroke slope of the early systolic shoulder is related to the left ventricular contractility whose abnormality can initiate a clinically significant heart failure syndrome [25, 26], the slope of the first peak as well as the magnitude ratio (radial AI) were evaluated to be in good agreement with the upstroke slope of the representative human waveform.

#### Radial augmentation index (AI)

**Fig. 10:**
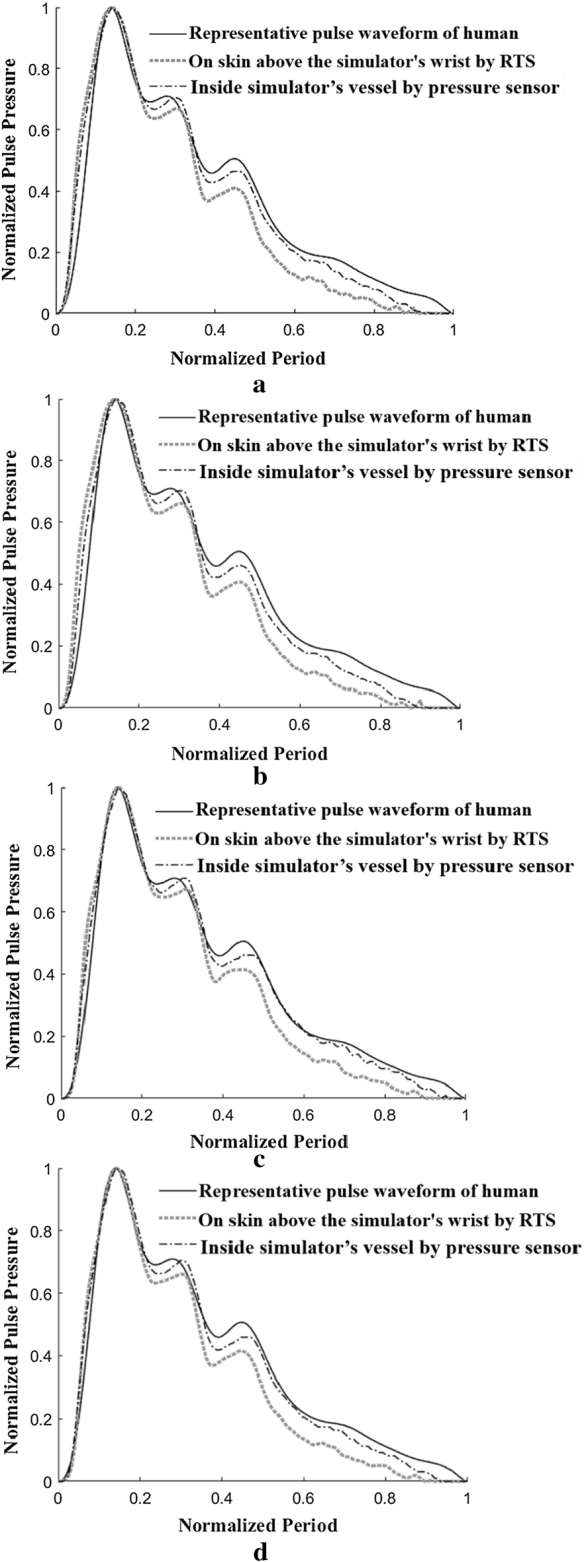
Comparison of pulse waveforms measured on a human’s wrist using RTS, on a simulator’s wrist using RTS and inside simulator’s vessel (tube) using a pressure sensor with varying heart rate and pulse pressure: **a** case with heart rate of 65 bpm and pulse pressure of 50 mmHg, **b** case with heart rate of 65 bpm and pulse pressure of 60 mmHg, **c** case with heart rate of 75 bpm and pulse pressure of 50 mmHg, **d** case with heart rate of 75 bpm and pulse pressure of 60 mmHg

For the various comparative analyses shown in Fig. 10, the heart rate was adjusted to 65 bpm and 75 bpm by changing the rotational speed of the built-in motor in the simulator. The pulse pressure was regulated to 50 mmHg and 60 mmHg, respectively, by adjusting the internal volume of the simulator. In each case, the measured pulses were stored using the RTS on the skin of the wrist of the simulator. At the same time, the pulses measured by a pressure sensor built into the simulator’s silicone vessel were stored. In each case, the average waveforms were generated from the stored pulses, and then the magnitude and period were normalized to 1 and compared with the representative waveform of a human (Fig. 4) as shown in Fig. 10.

Figure 10a shows the results obtained by measuring the heart rate and pulse pressure of the simulator at 65 bpm and 50 mmHg, respectively. In the figure, the measured data with the RTS and pressure sensor were compared with the representative waveform of a human. Figure 10b shows the comparison results when the simulator heart rate and pulse pressure were set to 65 bpm and 60 mmHg, respectively, and Fig. 10c shows the comparison results at 75 bpm and 50 mmHg. Figure 10d shows the comparison results at 75 bpm and 50 mmHg.

As shown in Fig. 10a, it was confirmed that the waveform measured by the RTS on the wrist of the simulator and the waveform measured by the pressure sensor are in good agreement with the human representative waveform. This result implies that the proposed three-peak cam generating the pressure waveform in the simulator is designed to accurately regenerate the human pulse waveform. As shown in Fig. 10b–d, similar trends were observed when the same comparisons were made by changing the heart rate and pulse rate.

**Fig. 11:**
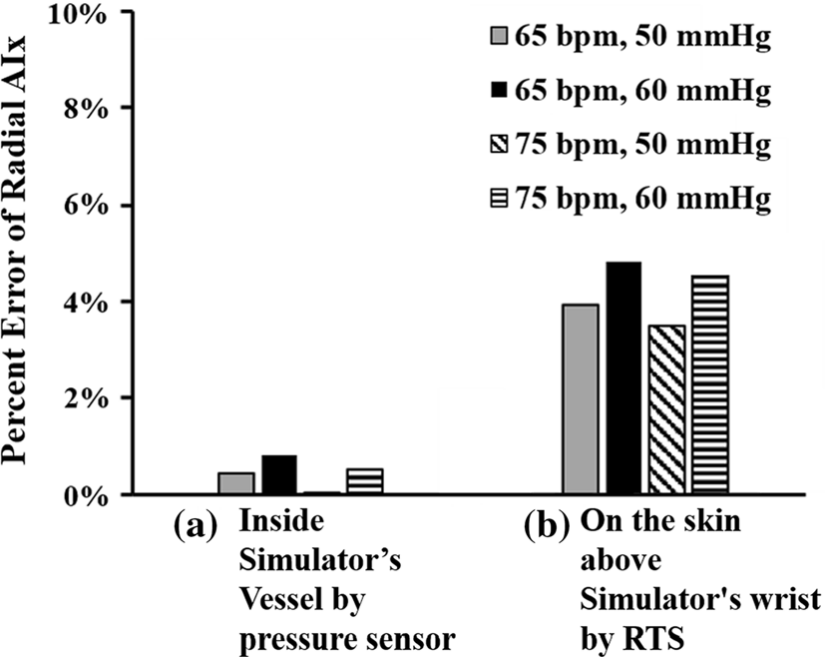
Error comparison of radial augmentation index: **a** error between measured data inside simulator’s vessel by a pressure sensor and human’s data, **b** error between measured data on simulator’s skin by RTS and human’s data

Figure 11a shows the error between the representative waveform of a human (Fig. 4) and the pressure waveform measured by a pressure sensor inside the simulator’s vessel in terms of the radial AI. Here, since the error value of the radial AI is very small at less than 8.14E−3, it was confirmed that the radial AI values of both waveforms were matched well. On the other hand, in Fig. 11b, the error value of the radial AI between the representative waveform of a human (Fig. 4) and the waveform measured by the RTS on the skin of the wrist of the simulator was about 4.85E−2, which is relatively large. The reason why the error value (waveform measured by the RTS) is relatively large is that the proposed simulator generates the pressure waveform using air pressure instead of an incompressible liquid similar to blood. Because the compressibility of air is different from that of blood, the pressure waveform measured by the tonometry method using the RTS has a slightly different radial AI value from that of the human radial AI. Although this error value is larger than the value in Fig. 11a, this error value is small enough to conclude that the developed simulator can reproduce a pulse waveform similar to the human waveform while ensuring the value of the radial AI.

### Phase Delay

As shown in Fig. 10, when comparing the upstroke slopes of early systolic pressure in the three measured waveforms, it can be seen that the slope of the representative pressure waveform of the human is the steepest. This is because the proposed simulator generates a pressure waveform by compressing and tensioning air instead of an incompressible liquid similar to blood. Owing to the nature of the air, which is a compressible fluid, the slope of the upstroke becomes less steep, resulting in a phase delay. To ensure that this phase delay effect is small enough to be ignored, an error analysis of the phase delay was performed among the representative pulse waveforms of a human, the pulse wave measured by the RTS on the skin above the simulator’s wrist, and the pressure sensor outputs built into the simulator’s vessel, as described in Fig. 10.

**Fig. 12:**
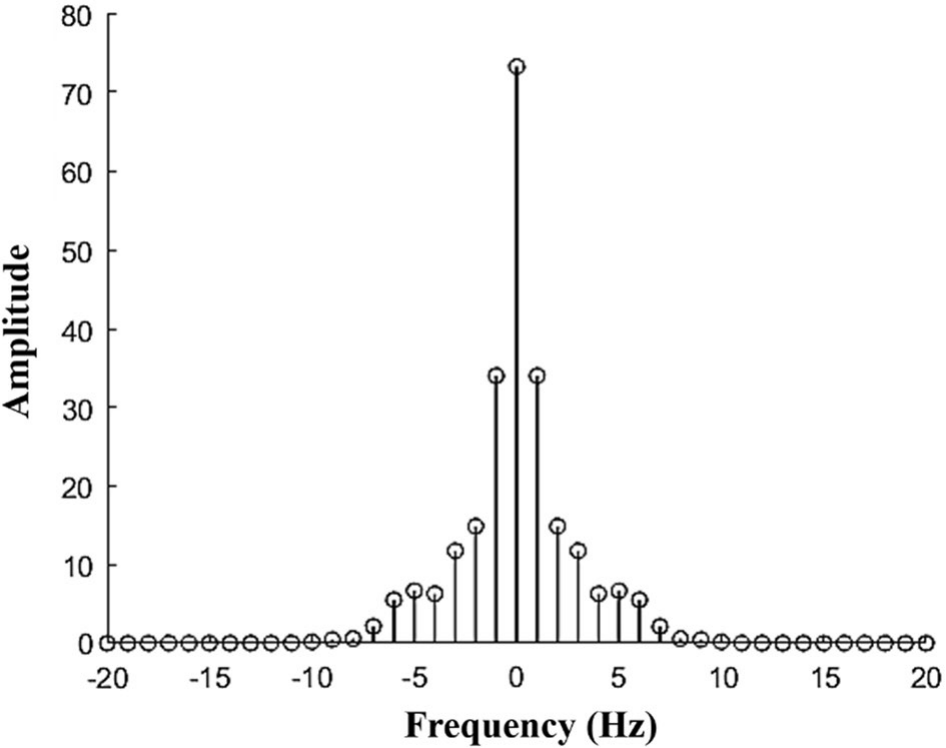
The result obtained by discrete Fourier transformation of the measured pressure waveforms in the human and the simulator

Figure 12 shows the amplitude of Fourier transform at each frequency in the frequency domain when a discrete Fourier transform was applied to the pulse waveforms measured in the human and the simulator. The results of the discrete Fourier transform showed almost no difference between the amplitudes obtained from the human waveform and simulator waveform. The results also showed that the pulse waveforms measured in the human and the simulator had a dominant amplitude in the low-frequency range. Thus, we investigated the difference in phase angle at low frequencies of 1 Hz and 2 Hz to determine the phase delay between pulse waveforms.

If $y^{h}$ is the pulse shape of a human, and $y^{c}$ is the waveform generated by the cam simulator, the discrete Fourier transform is defined as follows:

4 $$ \begin{aligned} \widehat{y}^{h} \left( f \right) = \mathop \sum \limits_{n = 1, \ldots ,N} y^{h} (x_{n} )e^{{ - \frac{2\pi i}{N}fn}} \hfill \\ \widehat{y}^{c} \left( f \right) = \mathop \sum \limits_{n = 1, \ldots ,N} y^{c} (x_{n} )e^{{ - \frac{2\pi i}{N}fn}} \hfill \\ \end{aligned} $$

Here, $x_{n} = x/N$ (N = 200). If $\widehat{y}^{h} \left( f \right)$ and $\widehat{y}^{c} \left( f \right)$ in Eq. (4) are expressed in the complex domain, the angles determined by the real parts and imaginary parts are denoted by $\theta \left( {\widehat{y}^{h} \left( f \right)} \right)$ and $\theta \left( {\widehat{y}^{c} \left( f \right)} \right)$, respectively. Here, the phase angle delay is defined as Eq. (5):

5 $$ Phase\;Angle \;Delay = \theta \left( {\widehat{y}^{h} \left( f \right)} \right) - \theta \left( {\widehat{y}^{c} \left( f \right)} \right) $$

At low frequencies of 1 Hz and 2 Hz, the phase angle delays are calculated as Eq. (6):

6 $$ \begin{aligned} Phase\;Angle \;Delay_{{\left( {f = 1Hz} \right)}} = \theta \left( {\widehat{y}^{h} \left( 1 \right)} \right) - \theta \left( {\widehat{y}^{c} \left( 1 \right)} \right) \hfill \\ Phase\; Angle\; Delay_{{\left( {f = 2Hz} \right)}} = \theta \left( {\widehat{y}^{h} \left( 2 \right)} \right) - \theta \left( {\widehat{y}^{c} \left( 2 \right)} \right) \hfill \\ \end{aligned} $$

**Fig. 13:**
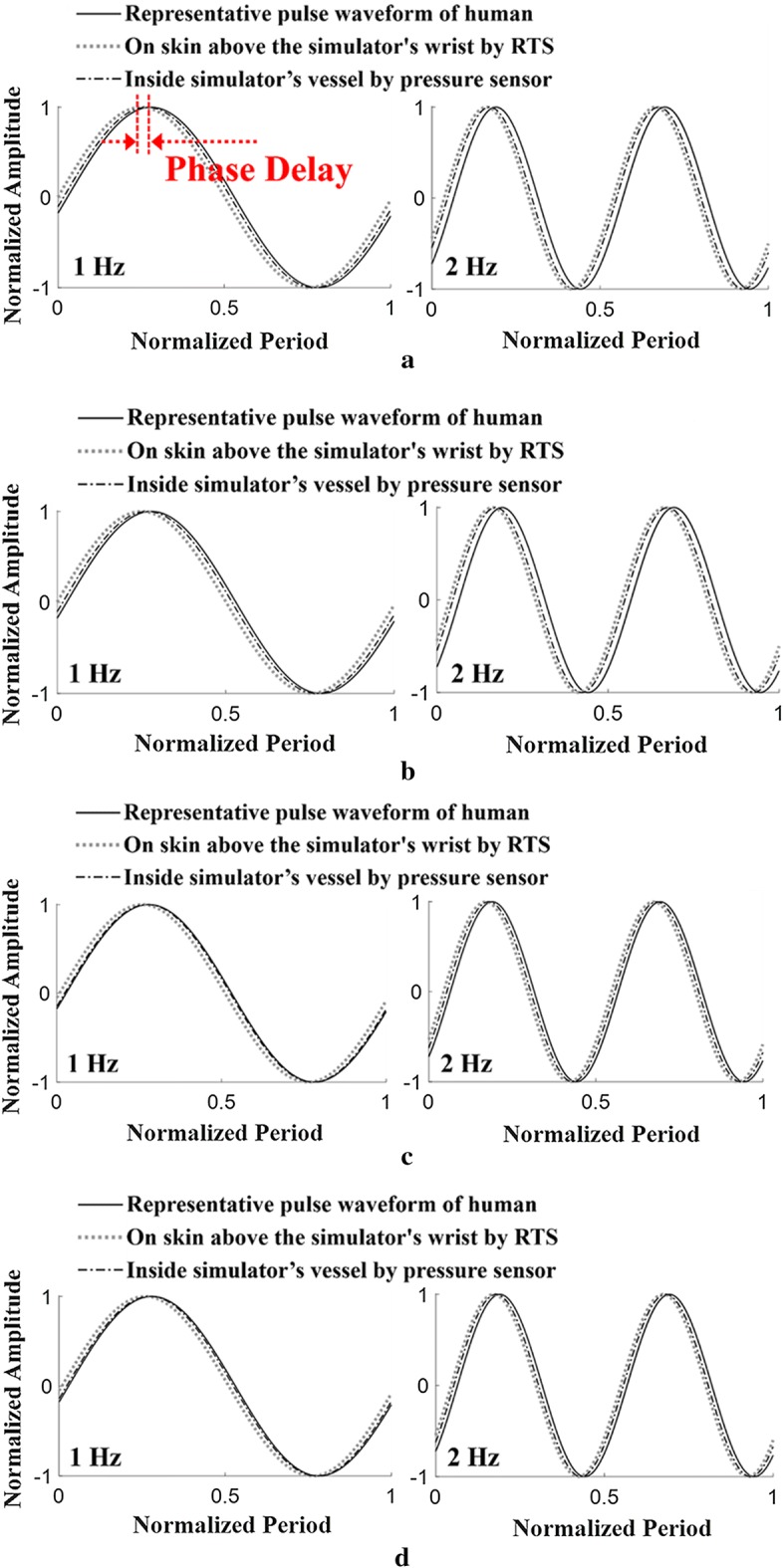
Phase delay comparison of waves of Fourier series of human pulse and pulse generated by CAM simulator at 1 Hz and 2 Hz: **a** case with heart rate of 65 bpm and pulse pressure of 50 mmHg, **b** case with heart rate of 65 bpm and pulse pressure of 60 mmHg, **c** case with heart rate of 75 bpm and pulse pressure of 50 mmHg, **d** case with heart rate of 75 bpm and pulse pressure of 60 mmHg

In four cases where the heart rate is adjusted to 65 bpm and 75 bpm and the pulse pressure is adjusted to 50 mmHg and 60 mmHg as shown in Fig. 10. Figure 13 illustrates the phase angle at $f = 1   {\text{Hz and }}f = 2   {\text{Hz}}$ of each pulse waveform measured in the human and the simulator. In all figures, the difference in phase angle is positive, indicating that the phase of the waveform is delayed.

**Fig. 14:**
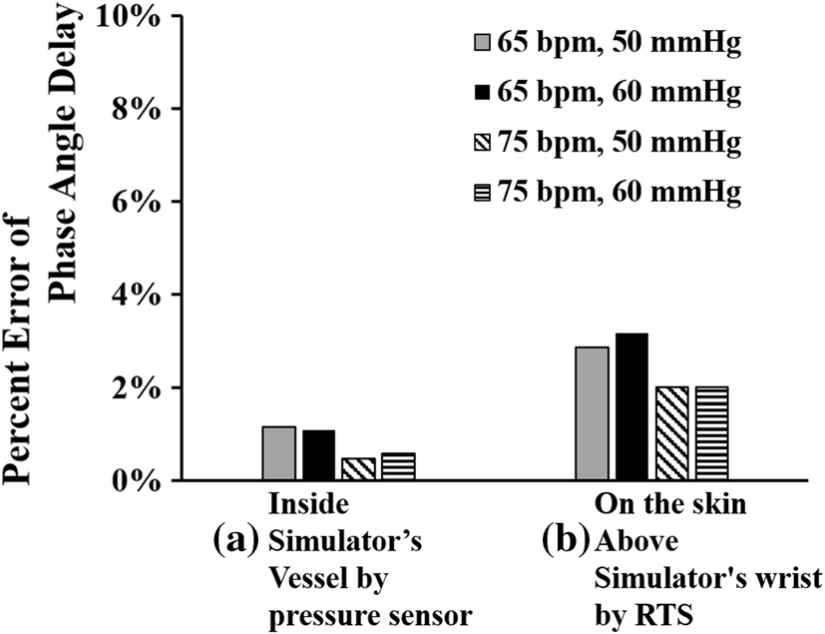
Error comparison of Fourier phase angle delay at 1 Hz: **a** error between measured data inside simulator’s vessel by a pressure sensor and human’s data, **b** error between measured data on simulator’s skin by RTS and human’s data

Figure 14 shows the error of the phase angle delay owing to heart rate and pulse pressure changes. The error value of the phase delay of the waveform measured on the skin of the simulator (Fig. 14b) is larger than the error value of the phase delay of the waveform measured by the pressure sensor (Fig. 14a). The reason is that the proposed simulator produces a pressure waveform using air pressure with compressibility characteristics, which results in a decrease in the pressure transfer efficiency to the skin.

A maximum phase angle delay of 11.4° occurs at a heart rate of 65 bpm and a pulse pressure of 60 mmHg, as shown in Fig. 14b. This phase angle delay is small enough to be 3.2% when converted to a percentage error in one cycle (360°). As a result, the phase delay effect caused by using air instead of incompressible liquid in the proposed simulator is sufficiently small, thus proving that a very accurate pulse pressure waveform can be reproduced using the air-based three-peak cam simulator.

## Conclusion

In this study, a radial pulsation simulator equipped with a cam mechanism was developed and tested. The developed simulator employed a pneumatic-driven mechanism to avoid the problems of liquid-driven devices, such as sporadic reflections of pressure waves, bubbles, and leakage. To design the cam profile, human pulse waveforms measured by a robotic tonometry system were mathematically modeled as one representative waveform. The representative waveform for a 20-year old was then converted into the circular cam profile. A cam design with three peak points was machined and mounted on a simulator, consisting of a rotating motor, a cylinder/piston module, an artificial wrist, and an LCD display module. The experimental results show that the proposed cam simulator can reproduce human representative waveforms with considerably small errors for the radial augmentation index and the phase delay effect with a maximum of 4.9% and 3.2%, respectively.

In summary, this study successfully developed a radial pulsation simulator based on a cam mechanism, and it demonstrated that the prototype simulator can accurately reproduce and control radial pulse waveforms, contributing to the advancement of a radial simulator that is cost-effective, portable, and reliable. Further work will be focusing on establishing representative radial pulse waveforms according to ages, race, and health conditions, and fabricating a cam with multiple peaks. Moreover, applications of the radial pulsation simulators equipped with a multiple-peak cam will be explored, including the verification of the computational simulation of blood flow, evaluation of wearable pressure sensors, studying the transfer functions or relationships between the radial and central pulse pressures, and training the pulse diagnosis of oriental medicine.
